# Ketogenic Metabolic Therapy for Glioma

**DOI:** 10.7759/cureus.26457

**Published:** 2022-06-30

**Authors:** Kris A Smith, Benjamin K Hendricks, Joseph D DiDomenico, Beth N Conway, Tracy L Smith, Amir Azadi, Ekokobe Fonkem

**Affiliations:** 1 Neurosurgery, Barrow Neurological Institute, Phoenix, USA; 2 Nutrition, Custom Nutrition Consultants, Phoenix, USA; 3 Neurology, Barrow Neurological Institute, Phoenix, USA

**Keywords:** metabolism, ketosis, ketones, high-grade glioma, glioblastoma

## Abstract

Purpose: This study describes a retrospective case series of patients with glioma who received ketogenic metabolic therapy through dietary adherence and intermittent fasting.

Methods: A retrospective chart review of a single surgeon’s clinic records was performed to identify patients who maintained nutritional ketosis for at least four months between January 2015 and October 2020.

Results: Sixteen patients who met the inclusion criteria constituted a heterogeneous population of patients with diagnoses including eight World Health Organization (WHO) grade IV gliomas (seven glioblastoma, one gliosarcoma), seven WHO grade III gliomas (three oligodendroglioma, four astrocytoma), and one WHO grade II oligodendroglioma. *IDH1* mutation status was present for 12 patients, and *MGMT* methylation status was present for eight patients. The mean (standard deviation [SD]) duration of ketogenic metabolic therapy was 20.6 (13.8) months. The Response Assessment in Neuro-oncology Criteria was applied during the ketogenic metabolic therapy interval, indicating a complete response in eight patients and partial response in eight patients. The mean (SD) progression-free survival while patients maintained ketogenic metabolic therapy was 20.0 (14.4) months.

Conclusion: Ketogenic metabolic therapy appears to convey a survival advantage within this patient series, which highlights the possibility that this therapy, when strictly applied, can augment the standard of care. Further exploration of this modality in a prospective series is warranted to formally explore this therapy.

## Introduction

High-grade glioma has remained resistant to standard medical therapies despite multiple advances in surgical, radiation, and chemotherapeutic options over the past few decades. Average survival for patients with treated glioblastoma (GBM) has been remarkably stable (approximately 12-16 months), with less than 2% of patients surviving more than five years [1]. Furthermore, patients who are long-term survivors of this disease with standard therapy experience significant cognitive and quality-of-life compromises. Most long-term survivors are reported to have treatment-induced dementia [2]. In 2016, the World Health Organization (WHO) revised the diagnostic criteria to differentiate an oligodendroglioma from an astrocytoma by requiring that 1p/19q co-deletion status be recognized as the requirement for oligodendroglioma diagnosis [3]. This criterion is clinically relevant given the significant survival advantage associated with an anaplastic oligodendroglioma diagnosis (five-year survival, 69%), compared with an anaplastic astrocytoma diagnosis (two-year average survival) [2,4].

Alterations in the metabolism of cancer cells were noted nearly 100 years ago by Dr. Otto Warburg [5]. Warburg won the Nobel Prize in 1931 for unraveling the oxygen-transferring ferment of respiration [6]. He had become obsessed with the altered metabolism of cancer cells as early as 1924 and published numerous papers on his theory on the metabolic origins of cancer cells, including seminal papers in Science in 1959 [7]. His theory was hotly debated and not widely accepted at the time; however, his insights have nearly been forgotten over the past century. Increased glycolysis and diminished tricarboxylic acid cycle activity and oxidative phosphorylation are seen very early in tumorigenesis and constitute one of the hallmarks of cancer [8]. Cancer cells use up to 18 times as much glucose as normal cells, because cancer cells use the inefficient metabolic process of fermentation (even in the presence of oxygen), as opposed to aerobic respiration, to produce adenosine triphosphate; this difference between cancer cells and healthy cells can be observed on positron emission tomography scans using fluorodeoxyglucose [9]. In 2011, Hanahan and Weinberg [10] extended the list of cancer hallmarks to include metabolic reprogramming, or deregulated cellular energetics, as an emerging hallmark and a potential cancer target. Recently, researchers have a newfound interest in attempting to exploit the need of cancer cells for excess glucose, compared with normal cells, as a therapeutic target [11,12].

Ketogenic metabolic therapy (KMT), a term coined in a review of six papers on the ketogenic diet in gliomas by Winter et al. in 2017 [13], is administered by prescribing variations of diets that are high in fat and low in carbohydrates with moderate protein intake to produce a naturally occurring state of nutritional ketosis [14]. Specific nutritional genomic variations between individuals explain why some people more easily obtain a state of nutritional ketosis compared with others [15]. Intermittent fasting is used for some patients as a means of increasing the degree and consistency of nutritional ketosis. Nutritional ketosis is defined by blood ketone concentrations greater than 0.5 mM [16,17], whereas “therapeutic ketosis” is considered more effective when the concentration of ketones in the blood is between 3.0 and 6.0 mM [18].

In the simplest understanding, depriving cancer cells of glucose with a strict low-carbohydrate diet should cause the cancer cells to be deprived of energy and become more stressed and susceptible to chemotherapy and radiation treatments [19]. Normal cells, and specifically neurons, function very well using ketones for fuel. Furthermore, being in a state of nutritional ketosis affords several favorable side effects in treating brain tumors, including decreasing edema by inhibiting the cyclooxygenase-2 pathway and by decreasing numerous inflammatory cytokines [19]. Laboratory animals with brain tumor model experiments treated with ketogenic formula had significantly less edema than their counterparts who were fed regular chow [20]. It was also noted that beta-hydroxybutyrate, the major ketone body in the bloodstream, acts as a histone deacetylase inhibitor [21]. This inhibition results in increased susceptibility of cancer cells to radiation and chemotherapy treatments by multiple epigenetic mechanisms [20-22]. Both in vitro and in vivo experiments have shown striking results when adding KMT to radiation and chemotherapy approaches for cancer cells in culture and in live animal brain tumor models [19-21]. Striking results were published by Zeng and colleagues [20], who showed that, in a murine GBM tumor model, cancer responded much better to treatment when the animals were fed a ketogenic diet. When animals were fed ketogenic formula (Ketocal; Nutricia, Zoetermeer, Netherlands) while receiving radiation and chemotherapy, 75% were completely cured of the GBM tumor implant and were proven to be tumor-free at one year when euthanized. Control animals fed normal chow lived an average of 18 weeks, and all died of tumor progression, with no animals surviving beyond 24 weeks [20]. Clinical applications of glioma treatment with KMT are limited [23]. In 2019, Woodhouse et al. [24] provided a retrospective analysis of 29 patients treated with a modified Atkins diet, in which all patients achieved ketosis during the study interval, demonstrating the feasibility and safety of maintaining ketosis during surveillance for gliomas. They analyzed survival only for patients who were at least two years from diagnosis; among patients with GBM (n=15), only four met the two-year survival criteria, which was consistent with existing non-KMT literature in which patients were treated using the standard of care. Hu et al. recently published a small phase 1 trial that showed safety and markedly favorable responses in some patients [25]. Despite these findings, no thorough studies have been published on KMT among patients with glioma.

These findings and anecdotal observation prompted a retrospective review of a single surgeon’s glioma patient population, selecting patients who reported adherence to a ketogenic diet as metabolic therapy for glioma. The resulting patient population is proposed to serve as preliminary evidence for metabolic therapy and to encourage rigorous study in this promising frontier for glioma management.

## Materials and methods

Patient selection

A single surgeon’s clinic record for patients who received a diagnosis of glioma was analyzed, and patients were screened for age greater than 18 years from January 2015 to October 2020. Institutional review board approval for this study was obtained through the Barrow Neurological Clinical Outcomes Database. The St. Joseph’s Hospital and Medical Center Institutional Review Board confirmed that no additional ethical approval was required for this retrospective observational study. Given the retrospective nature of this small patient series and the lack of disclosing patient health information, the project did not meet the definition of human subject research; therefore, retrospective patient informed consent for study analysis was not pursued.

All patients who received a diagnosis of glioma during the time interval above were presented with the option of KMT. Patients who did not have clinical documentation of reported adherence to nutritional ketosis (e.g., by blood serum ketometer readings) indicating that nutritional ketosis was maintained for at least four months during the postdiagnosis surveillance interval were excluded. The documentation had to indicate that ketometer readings were performed, not the ketometer values. All data for patients included in the study were collected and de-identified. Pathology reports were reviewed and confirmed that all patients received a diagnosis of cerebral glioma.

Diet description

Patients were not given a specific meal plan to follow but were instructed by a registered dietician on the theory and practice of the ketogenic diet (generically, switching from carbohydrate metabolism to fat metabolism and thereby producing ketone bodies). Several versions of the ketogenic diet are published [23]. We prescribed a primarily plant-based and high-fiber ketogenic diet. Criticisms of the standard ketogenic diet include that it is low in fiber and can result in constipation and that it provides limited food choices. We advised patients to maximize eating green leafy vegetables, focus on tracking net carbohydrates, and disregard the consumption of fiber carbohydrates. Patients were encouraged to use olive oil, grass-fed butter, medium-chain triglycerides, and coconut oils and to avoid processed vegetable oils, trans fats, and fried foods. Patients were told to avoid grains, bread, pasta, potatoes, corn, and tropical fruits. Berries were allowed in limited quantities, and the total number of net carbohydrates was to be kept below 20 g per day. Intermittent fasting with a limited eating window of less than eight to 10 hours per day was reported by several patients. Blood serum-based ketometers were used to assure nutritional ketosis via both at-home (patient-reported) and in-clinic measurements, but the frequency of ketometer testing varied substantially.

Radiographic surveillance

Preoperative, postoperative, and interval surveillance magnetic resonance imaging was independently reviewed. Tumor progression was defined by the progressive enlargement of T1-weighted contrast-enhancing regions. Consideration was given to the progression of cerebral edema as observed on T2-weighted fluid-attenuated inversion recovery (FLAIR) image sequences. Response Assessment in Neuro-oncology (RANO) criteria were applied to judge response to therapy and progression when appropriate within the study. Radiographic follow-up frequency varied within the study population and was determined based on the preferences of the neurosurgeon, neuro-oncologist, and radiation oncologist.

Data analysis

Data were reported as the number of patients with characteristics and mean (standard deviation [SD]) for continuous variables. SPSS Statistics version 25 (IBM Corp., Armonk, NY) was used to manage and analyze data.

## Results

Sixteen patients met the inclusion criteria (Table 1); 10 were men and six were women.

**Table 1:** Demographic and clinical characteristics of 16 patients with glioblastomas who received KMT Astro, astrocytoma; BCNU, carmustine wafers; BVZ, bevacizumab; Dx, diagnosis; FU, follow-up; GBM, glioblastoma; GT, GammaTile; IMRT, intensity-modulated radiation therapy; KMT, ketogenic metabolic therapy; KPS, Karnofsky Performance Status; LITT, laser interstitial thermal therapy; NA, not available; oligo, oligodendroglioma; PFS, progression-free survival; Pt., patient; RANO, Response Assessment in Neuro-oncology; SRS, stereotactic radiosurgery; TMZ, temozolomide; TTF, tumor-treating fields; WHO, World Health Organization.

Pt.	Age at Dx, yr/Sex	Pathologic WHO Grade (Grade at Original Dx)	Pathologic Subtype	IDH1 Mutant	MGMT	Duration of KMT, mo.	Ketone Level, mM	Intermittent Fasting	RANO Response During KMT	PFS With KMT, mo.	Radiographic Progression During KMT	Radiographic Progression After KMT Cessation	Adjuvant Treatment	Last FU KPS
1	36/M	III	Oligo	+	-	36	1.5-4.5	+	Partial	18	-	Yes	TMZ, BVZ, GT, BCNU, LITT, SRS, TTF	0
2	42/F	IV (III)	Oligo	+	+	12	1.0	+	Complete	6	Yes	-	TMZ, IMRT, BVZ, GT, BCNU, TTF	0
3	59/F	III	Astro	+	+	36	0.5-1.8	-	Complete	34	No	-	TMZ, IMRT	90
4	25/M	IV (II)	Oligo-astro	+	+	12	NA	-	Partial	8	Yes	-	TMZ, IMRT, GT, BCNU, LITT, TTF	0
5	58/M	IV	GBM	NA	+	3	1.0	-	Partial	3	No	-	TMZ, IMRT, LITT	90
6	50/M	III	Astro	+	+	12	NA	-	Partial	12	No	-	TMZ, IMRT, LITT, TTF	90
7	56/M	IV (II)	Oligo with sarcomatous features	+	NA	7	0.5-1.0	+	Partial	7	-	Yes	TMZ, IMRT, BVZ, BCNU, LITT, SRS, TTF	20
8	75/M	IV	GBM	-	NA	6	1.5	NA	Partial	6	No	-	TMZ, IMRT, TTF	90
9	53/M	IV	Glio­sarcoma	-	NA	12	NA	+	Partial	12	-	Yes	TMZ, IMRT	0
10	35/F	IV	GBM	+	+	20	0.5-2.0	+	Partial	20	-	Yes	TMZ, IMRT, BVZ, GT, BCNU, SRS, TTF	80
11	44/M	II	Oligo	+	+	48	0.2-1.0	-	Complete	48	No	-		90
12	48/M	III	Oligo	+	NA	12	0.2-2.5	-	Complete	12	No	-	TMZ, IMRT, GT, LITT, SRS	90
13	27/M	III	Astro	+	-	24	4.0-5.0	+	Partial	24	No	-	TMZ, IMRT, TTF	100
14	53/M	III	Astro	-	-	12	1.0-3.5	+	Progression	0	Yes	-	TMZ, IMRT, BVZ, GT, LITT, TTF	80
15	54/F	IV	GBM	+	+	22	0.3-1.0	-	Partial	22	No	-	TMZ, IMRT, BVZ, BCNU, LITT, TTF	70
16	57/F	III (II)	Oligo	+	NA	3	NA	-	Partial	3	No	-	TMZ, IMRT, LITT, SRS	90

Their mean (SD) age at the time of diagnosis was 48.3 (4.8) years. The population was substantially heterogeneous, with diagnoses ranging from low-grade glioma (WHO grade II) to GBM (WHO grade IV). In our cohort, the diagnoses at the time of KMT initiation included eight WHO grade IV gliomas (four GBM, one gliosarcoma, and three grade IV oligodendroglioma), seven WHO grade III gliomas (three oligodendroglioma, four astrocytoma), and one WHO grade II oligodendroglioma. IDH1 mutation status was present for 12 patients, and MGMT methylation status was present for eight patients.

Most patients were salvage cases and had already undergone extensive therapy (Table 1) but were experiencing tumor progression; these patients experienced complete or near-complete cessation of tumor progression after KMT initiation. Two patients (patients 9 and 10) experienced a significant therapeutic response to KMT over several months but later experienced disease progression when not adhering to the diet. One patient (patient 4) initially demonstrated a significant response to KMT but later experienced progression of disease despite maintaining some degree of ketosis. One patient (patient 14) who initiated and maintained KMT initially had a radiographic response but later experienced disease progression. The series identified a case (patient 11) wherein focal enhancement (presumed malignant degeneration) within a low-grade glioma resolved completely, thereby thwarting tumor progression without radiation or chemotherapy after the adoption of KMT.

Among our 16 patients, the mean (SD) duration of KMT was 20.6 (13.9) months. The RANO response was complete in eight patients and partial in eight patients. The mean duration of progression-free survival while patients maintained KMT was 20.0 (14.4) months. Serum beta-hydroxybutyrate levels were assessed; however, these were inconsistently documented in the electronic medical record, and the frequency of testing varied. Values were available for 12 patients, with a mean value of 1.6 (1.1) mM during the KMT interval; intermittent fasting was used by seven patients.

Sixteen of 16 patients (100%) demonstrated significant improvement in radiographic appearance and clinical status after the initiation of KMT when combined with other, more standard adjuvant treatments. No patients reported unexpected adverse effects of KMT. On the contrary, many of the patients reported overall improvements in subjective quality-of-life assessments using the 36-item Short Form Survey during the KMT treatment interval. These quality-of-life assessments were not consistently available and, therefore, were not included in the data set for analysis. The two patients with the longest continuous KMT both had a widely diffuse and multifocal disease and had remarkable resolution of both imaging abnormalities, including enhancing nodules, which was maintained for more than three years of KMT.

Four patients voluntarily terminated KMT (patients 1, 7, 9, and 10). Each of those four patients terminated KMT after demonstrating a favorable response and experienced deterioration in clinical status and demonstrated radiographic progression requiring salvage treatment within two to four months of KMT cessation. Patient 9 died after KMT cessation and disease progression; this outcome was related to poor access to care. Patients 1 and 7 demonstrated a recurrent favorable radiographic response with decreased tumor burden and improved clinical status after resuming KMT. Unfortunately, patient 10 continued to experience disease progression despite a resumption of KMT.

Radiographic progression was noted in three patients who reliably maintained KMT through the surveillance interval (patients 2, 4, and 14). Patient 14 initially demonstrated a response to KMT while demonstrating consistent ketosis over a 12-month interval but at eight months required salvage surgery and additional treatments. After an initial favorable response during six and eight months of KMT, patients 2 and 4, respectively, experienced progression of the disease and died. The remaining nine patients maintained KMT through the most recent clinical follow-up, experienced complete or partial RANO responses, and had no radiographic disease progression during the study period.

Case example 1 (patient 3)

**Figure 1:**
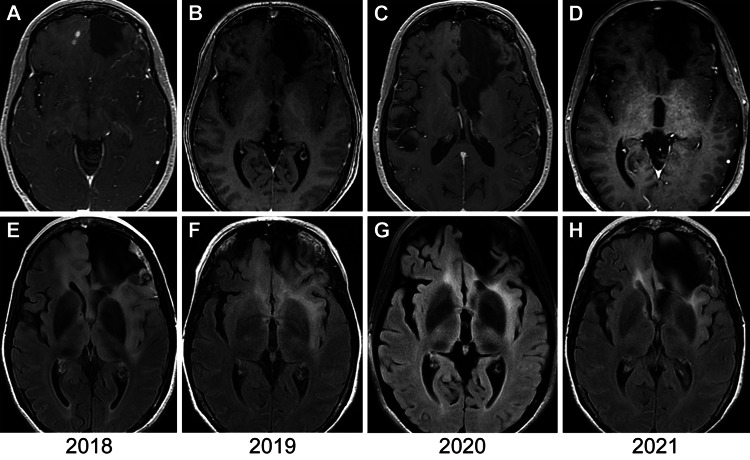
MRIs for patient 3 Axial magnetic resonance imaging (MRI) for patient 3 showing multiple enhancing nodules at the time of recurrence (2018) within the right frontal lobe (A). Annual follow-up T1-enhanced axial MRIs from 2019 (B), 2020 (C), and 2021 (D), obtained during 36 months of continuous ketogenic metabolic therapy, show that the patient experienced complete resolution of tumor enhancement. The patient demonstrated increased fluid-attenuated inversion recovery (FLAIR) signal intensity at the time of recurrence (2018) (E), with progressive improvement in FLAIR signal intensity and distribution in 2019 (F), 2020 (G), and 2021 (H). Used with permission from Barrow Neurological Institute, Phoenix, Arizona.

Patient 3 was a woman in her late 50s with left frontal anaplastic astrocytoma, with IDH1 mutant and MGMT methylated status, who underwent initial resection in 2004, and was treated with radiotherapy and temozolomide therapy; she underwent a research protocol treatment, including stereotactic radiosurgery and gefitinib therapy, in 2005. She lacked disease progression for more than 10 years. However, she demonstrated a multifocal bilateral recurrence in 2017 with increased cerebral edema and multiple enhancing nodules in 2018 (Figure 1A), with associated cerebral edema (Figure 1E).

Given the contraindication to right frontal lobectomy, KMT was recommended. The patient was prescribed TMZ for one year starting in 2017 and then fully implemented the ketogenic diet in November 2018. She has maintained ketosis for the subsequent 36 months at the time of this writing. Follow-up magnetic resonance imaging at three- to four-month intervals initially showed no disease progression and then the complete resolution of enhancement (Figure 1B-1D) and improvement in the FLAIR intensity (Figure 1F-1H). The patient maintained KMT at the last follow-up and continued to function well with no additional therapies. She experienced a healthy weight loss of approximately 18 kg and described the improved quality of life with respect to increased overall physical activity and mental clarity since changing her diet.

Case example 2 (patient 10)

**Figure 2:**
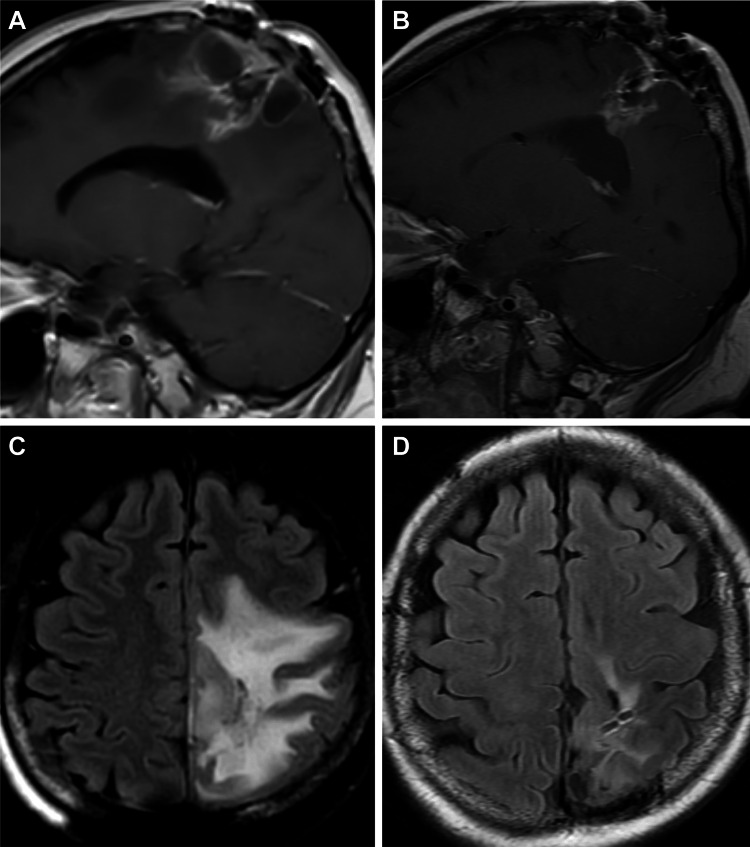
MRIs for patient 10 Patient 10 had continued disease progression after a second surgical resection as evidenced by increasingly nodular enhancement (A, sagittal magnetic resonance imaging [MRI]) and progressive cerebral edema (B, axial MRI). The patient subsequently initiated ketogenic metabolic therapy, and imaging obtained at the next follow-up evaluation (four months later) showed significant improvement in lesional enhancement (C, sagittal MRI) and markedly reduced cerebral edema (D, axial MRI). Used with permission from Barrow Neurological Institute, Phoenix, Arizona.

Patient 10 was a woman in her mid-30s who received a diagnosis of GBM in 2014 after surgical resection, which was determined to be IDH1 mutated and MGMT methylated. Subsequent therapy with radiation and temozolomide was given in a standard fashion. She demonstrated disease recurrence in late 2017 and was treated with additional surgical resection, implantation of cesium-131, and reinitiation of therapy with temozolomide. During the follow-up interval, nodular enhancement (Figure 2A) and progressive cerebral edema (Figure 2B) developed.

At this time, the patient started KMT and thereafter noted subjective improvement in her functional status as well as objective improvement in strength. Her subsequent follow-up magnetic resonance imaging findings showed significant improvement in lesional enhancement (Figure 2C) and cerebral edema (Figure 2D), which permitted her to taper steroid therapy completely for the first time in eight months. She terminated KMT after 20 months of adherence; four months later, radiographic and clinical progression was evident, which required additional surgical resection. The patient then initiated treatment with tumor-treating fields (TTFs) (Optune; Novocure Ltd., Saint Helier, Jersey) and received additional adjuvants including CyberKnife radiosurgery. Despite resuming adherence to KMT, the patient had not experienced an improvement in her clinical or radiographic disease state at the most recent follow-up.

Case example 3 (patient 1)

**Figure 3:**
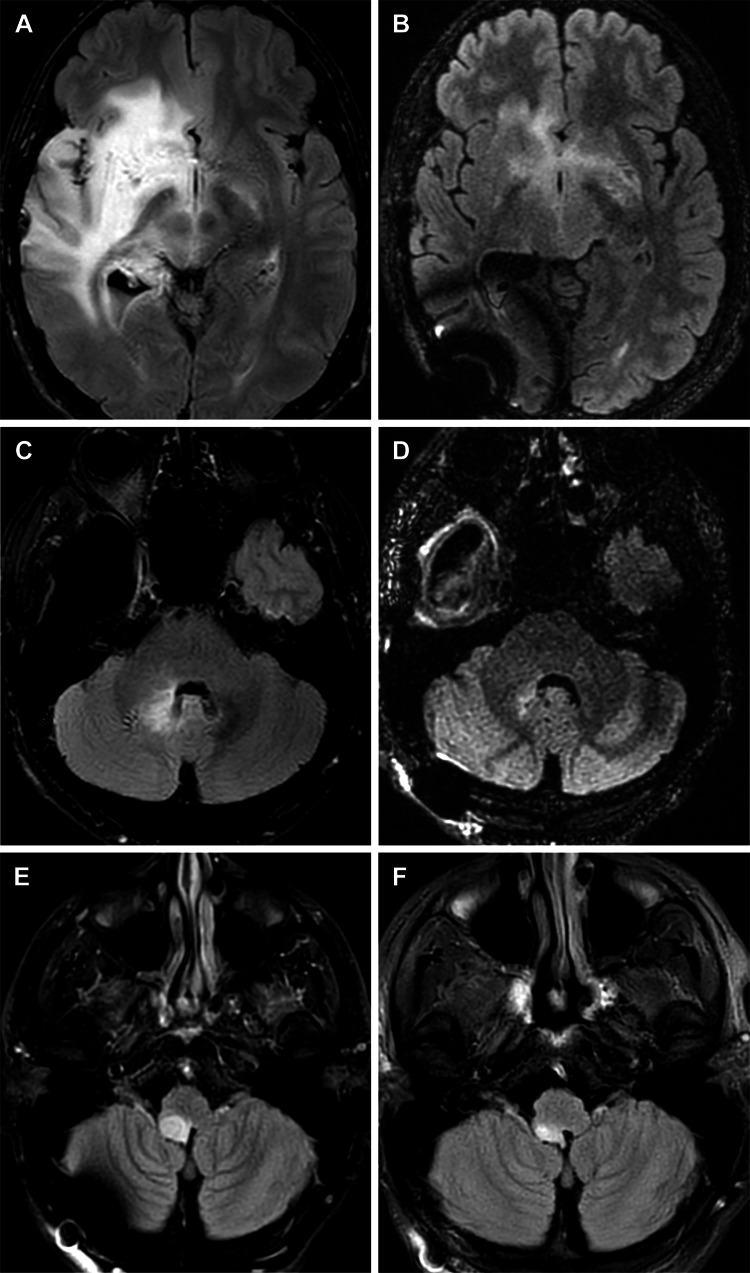
MRIs for patient 1 Axial magnetic resonance images (MRIs) for patient 1, who underwent repeat surgical resection, show that afterward the patient had continued progression of the tumor within the right insula, thalamus, basal ganglia, basal forebrain (A), and cerebellomedullary region (C). After initiation of ketogenic metabolic therapy, tumor-treating fields therapy, and temozolomide therapy, imaging shows near-complete resolution of the diffuse multifocal fluid-attenuated inversion recovery anomaly in the right insula, thalamus, basal ganglia, basal forebrain (B), and cerebellomedullary region (D). After a period of time during which the patient did not achieve ketosis, subsequent imaging showed progression of the tumor within the right medial temporal lobe and right medulla (E). Metabolic genomic profiling identified the cause of the patient’s inability to achieve ketosis, and he reinitiated ketogenic metabolic therapy. Because of its severity, the cerebellomedullary lesion was treated with Zap-X stereotactic radiosurgery. Follow-up imaging showed that the size of the lesion was greatly reduced (F). Used with permission from Barrow Neurological Institute, Phoenix, Arizona.

Patient 1 was a man in his mid-30s who received an initial diagnosis of WHO grade II oligodendroglioma with IDH mutated and MGMT unmethylated status in March 2016. The patient underwent subtotal resection but declined radiotherapy and temozolomide therapy. He experienced disease progression to WHO grade III anaplastic oligodendroglioma, which was resected in February 2018, and he had an implant of cesium 131 seeds in a clinical trial. The patient subsequently experienced progressive diffuse multifocal disease (nonenhancing FLAIR anomaly) in the insula, thalamus, basal ganglia, and forebrain as well as the cerebellomedullary region (Figures 3A, 3C).

Following a presentation of options, the patient elected for concurrent KMT (with intermittent fasting), temozolomide therapy, and TTFs in hopes of avoiding wide-field radiotherapy. At subsequent follow-up, the patient had near-complete resolution of diffuse multifocal FLAIR anomalies (Figures 3B, 3D) and reported subjective improvement in his functional status. Of interest, the patient was unable to maintain therapeutic ketosis while not fasting because of two discovered genetic polymorphisms (PPAR-a, SLC-2285), which made it necessary to further alter his dietary approach. While not in ketosis, he gained approximately 10 kg but also developed two other foci of progressive tumor in the right posterior medial temporal lobe and right medulla (Figure 3E). The patient underwent resection plus cesium brachytherapy for treatment of the temporal lesions. During follow-up, the patient became progressively symptomatic, with gait ataxia and imbalance, and required salvage treatment with hypofractionated Zap-X radiosurgery for the medullary lesion. Fortunately, with increased dietary manipulation based on nutrition genomics insights, the patient was again able to maintain therapeutic ketosis with ketone levels consistently in the 2.5-3.8 mM range. He had a remarkable response to the radiosurgery treatment of the medullary lesion, experienced complete resolution of the ataxic symptoms, and did not require subsequent steroid treatment (Figure 3F). With this history of multifocal recurrences, the tumor board recommended systemic therapy, and the patient initiated an IDH-1 blocker (ivosidenib) in March 2021. The patient experienced multifocal progression, including intraventricular and leptomeningeal disease that was extensive and included multifocal spinal involvement. His condition failed to respond to intrathecal chemotherapy, and he was recommended for hospice care and died in September 2021.

## Discussion

The standard of care for patients with high-grade gliomas has continued to provide very disappointing results with respect to both tumor control and quality of life. The most promising recent results in a large trial were reported when TTFs were added to the Stupp protocol in a large European trial [26]. The median survival of the group treated with TTFs increased to 20.9 months compared with 16 months in the standard cohort. However, the median duration of progression-free survival was only 6.7 months in the TTF group and only 4.0 months in the group that received standard care. A better understanding of the underlying disease process is needed to arrive at a more effective treatment strategy. Synergistic drug combinations combined with precision-medicine approaches have been suggested, with an understanding of the multiple mechanisms of resistance to the standards of care [27].

Temozolomide therapy has been shown to strongly suppress proinflammatory cytokines and escape the immune response as a mechanism of resistance and later tumor recurrence [27]. Polo-like kinase 4 was also shown to be markedly upregulated in some GBMs as a mechanism of radiation resistance and was associated with an even worse prognosis with standard care. Ma et al. [28] reported that the FoxM1/BUB1B signaling pathway was implicated in tumorigenesis and radiation resistance in some GBMs and was also associated with a worse-than-normal prognosis when detected with molecular profiling.

These are only a sampling of the many molecular aberrations and variations in high-grade gliomas that result in resistance to standard of care and a poor prognosis for almost all patients. We, therefore, propose that reconsidering the potential of the metabolic underpinnings of the disease may lead to a more effective combination of treatment options.

Although our findings are preliminary, a mean (SD) progression-free survival of 13.3 months (8.4) months among patients with GBMs receiving KMT (n=8) and 17.5 (13.8) months among patients with WHO grade III glioma (n=7) is suggestive of a significant role for KMT in the treatment of high-grade glioma. These results are favorable to other interventions for recurrent high-grade gliomas. This finding warrants further prospective analysis in a larger patient population. The failure of patient 10 to achieve a response after reinitiating KMT following a cessation interval was suggestive of a certain adaptation, possibly a conversion to glutamine fermentation, after which KMT efforts may become futile.

The finding of both PPAR-a and SLC-22A5 polymorphisms in patient 1 posed a surprising challenge for maintaining KMT in that patient. PPAR-a is a transcription factor activated by fatty acids that plays a critical role in the starvation response. Fatty acid-bound PPAR-a imparts downstream molecular changes that impact anti-inflammatory actions and cellular metabolic enzymes [29,30]. This patient’s gene variant, RS 1800206 CG, is associated with increased low-density lipoprotein cholesterol, decreased high-density lipoprotein cholesterol, and increased triglycerides and C-reactive protein compared to the normal gene variant. He was able to maintain therapeutic ketosis while in prolonged fasts but struggled to have measurable ketones in response to a low-carbohydrate, high-fat diet without time-restricted eating, presumably due to this variant.

SLC-22A5 is an important protein family within the solute carrier superfamily, which is critical within the digestive system due to its role in oxidative phosphorylation [31]. These carrier proteins shuttle the substrates for metabolism along the mitochondrial membrane. Many well-described rare mitochondrial diseases result from gene mutations severe enough to cause complete loss of function of these carrier proteins [31]. It is common to have less-severe single-nucleotide polymorphisms of some of these genes, which nevertheless cause some less-noticeable pathologies. Patient 1 had two homozygous variants (RS17622208 and RS2073643) that could have impacted carnitine transport and was, therefore, given L-carnitine supplementation. He appeared to respond to this as evidenced by increased blood ketone levels following supplementation. The markedly negative response to an IDH-1 blocker in this patient with mutant IDH-1 was unexpected and tragic for this patient. It is unknown if this agent contributed to widespread leptomeningeal dissemination; however, it was clearly not helpful for his case, and caution for other patients is warranted until further data are available.

The weaknesses of this study are extensive given the variety of pathological subtypes of glioma diagnosed and the variations in other therapies used within the limited sample of 16 patients. The differing stages of progression at the time of initiating KMT and the lack of consistent adherence to KMT within the study population also significantly affect the reliability of the findings. The patients also received a multitude of therapeutic interventions within the surveillance interval, such as repeat resection, laser interstitial thermal therapy, TTFs, adjuvant radiotherapy, salvage chemotherapy, and radiosurgery and brachytherapy. The application of these multiple treatment modalities resulted in a heterogeneous patient population, which further limits the strength of the evidence. Importantly, given the application of concurrent therapies, it is difficult to attribute any radiographic response to KMT directly versus the other treatment modalities these patients received.

## Conclusions

Despite the limitations of this study, KMT appeared to convey a substantial survival advantage to those patients who diligently adhered to the dietary regimen, highlighting that this therapy, when strictly applied, holds promise as a therapeutic intervention. The radiographic and clinical responses of some of these patients were truly extraordinary. The difference in response to KMT evident within the series is suggestive of the potential value for nutritional genomics evaluation of patients for optimization of KMT, which would personalize and enhance the delivery of this therapy. The preliminary evidence in this report informs the literature of the role KMT can play for patients with glioma and should prompt an extensive, prospective, quantitative, controlled study of this therapy.
